# All Treatment Parameters Affect Environmental Surface Sanitation Efficacy, but Their Relative Importance Depends on the Microbial Target

**DOI:** 10.1128/AEM.01748-20

**Published:** 2020-12-17

**Authors:** Shiyu Cai, David M. Phinney, Dennis R. Heldman, Abigail B. Snyder

**Affiliations:** a Department of Food Science, Cornell University, Ithaca, New York, USA; b Department of Food Science and Technology, The Ohio State University, Columbus, Ohio, USA; University of Manchester

**Keywords:** computational fluid dynamics, sanitation, spoilage, food safety

## Abstract

Environmental sanitation in food manufacturing plants promotes food safety and product microbial quality. However, the development of experimental models remains a challenge due to the complex nature of commercial cleaning processes, which include spraying water and sanitizer on equipment and structural surfaces within manufacturing space. Although simple in execution, the physical driving forces are difficult to simulate in a controlled laboratory environment. Here, we present a bench-scale bioreactor system which mimics the flow conditions in environmental sanitation programs. We applied computational fluid dynamic (CFD) simulations to obtain fluid flow parameters that better approximate and predict industrial outcomes. According to the CFD model, the local wall shear stress achieved on the target surface ranged from 0.015 to 5.00 Pa. Sanitation efficacy on six types of environmental surface materials (hydrophobicity, 57.59 to 88.61°; roughness, 2.2 to 11.9 μm) against two different microbial targets, the bacterial pathogen Listeria monocytogenes and *Exophiala* species spoilage fungi, were evaluated using the bench-scale bioreactor system. The relative reduction ranged from 0.0 to 0.82 for *Exophiala* spp., which corresponded to a 0.0 to 2.21 log CFU/coupon reduction, and the relative reduction ranged from 0.0 to 0.93 in L. monocytogenes which corresponded to a 0.0 to 6.19 log CFU/coupon reduction. Although most treatment parameters were considered statistically significant against either L. monocytogenes or *Exophiala* spp., contact time was ranked as the most important predictor for L. monocytogenes reduction. Shear stress contributed the most to *Exophiala* spp. removal on stainless steel and Buna-N rubber, while contact time was the most important factor on HDPE (high-density polyethylene), cement, and epoxy.

**IMPORTANCE** Commercial food manufacturers commonly employ a single sanitation program that addresses both bacterial pathogen and fungal spoilage microbiota, despite the fact that the two microbial targets respond differently to various environmental sanitation conditions. Comparison of outcome-based clusters of treatment combinations may facilitate the development of compensatory sanitation regimes where longer contact time or greater force are applied so that lower sanitizer concentrations can be used. Determination of microbiological outcomes related to sanitation program efficacy against a panel of treatment conditions allows food processors to balance tradeoffs between quality and safety with cost and waste stream management, as appropriate for their facility.

## Abstract (precis)

Commercial food manufacturers commonly employ a single sanitation program that addresses both bacterial pathogen and fungal spoilage microbiota, despite the fact that the two microbial targets respond differently to various environmental sanitation conditions. Comparison of outcome-based clusters of treatment combinations may facilitate the development of compensatory sanitation regimes where longer contact time or greater force are applied so that lower sanitizer concentrations can be used. Determination of microbiological outcomes related to sanitation program efficacy against a panel of treatment conditions allows food processors to balance tradeoffs between quality and safety with cost and waste stream management, as appropriate for their facility.

## INTRODUCTION

Cross-contamination from surfaces within the food production environment is increasingly recognized as an important source of food spoilage microbes and foodborne bacterial pathogens. Effective sanitation programs are the primary bulwark against environmental cross-contamination as referenced in the United States. U.S. Food and Drug Administration draft guidance on the control of Listeria monocytogenes in ready-to-eat foods (1). The fundamental parameters of any sanitization step are defined by (i) contact time, (ii) active chemical concentration, (iii) applied force, and (iv) treatment temperature (2). In environmental surface sanitation, manual cleaning and sanitizer application to food contact and non-food contact surfaces is based on a combination of chemical and mechanical action to achieve a desired level of surface hygiene. However, it is difficult to evaluate the microbiological outcomes of these programs due to the inherent complexity of sanitation, which is a function of both treatment and environmental surface factors. A model system that allows for controlled evaluation of individual sanitation parameters could help bridge the gap between experimental data and commercial systems. One of the challenges in development of a model system is incorporation of reproducible shear stress in bench-scale simulation of environmental sanitation activities.

Much previous work evaluating sanitizer efficacy has characterized inactivation on various food contact surfaces without accounting for the mediating impact of shear stress, a relevant variable in commercial application (3–7). Wall shear stress is the tangential force of the flowing cleaning solution on the soiled surface and has been identified as an essential parameter in sanitation effectiveness, but it is difficult to model experimentally and extrapolate to industrial settings. Computational fluid dynamics (CFD) is the numerical simulation of fluid motion. In the sanitation literature, CFD has previously been applied in the evaluation of sanitation in clean-in-place (CIP) systems (8–11). CFD has also been applied in evaluation of shear stress during processing operations (12, 13). In this study, we apply these advanced modeling tools in the simulation of environmental sanitation.

Sanitation regimes are used to control environmental cross-contamination from foodborne pathogens and spoilage biota which encompasses a range of microbial targets across the domains of *Bacteria* and *Eukarya* (e.g., yeast and molds). However, historically, much of the focus of environmental sanitation work has been on mitigation of bacterial pathogens, notably Listeria monocytogenes (3, 5, 14–24). Both pathogenic and spoilage bacteria, as well as spoilage fungi, are relevant targets and are likely to be affected differently by various treatment parameters of the sanitation program (25). Assessing the impact of sanitation treatments on various microbial targets, in addition to the evaluation of the mediating impact of microbial targets on one another, remains an important consideration in the optimization of sanitation programs. By determining the relative efficacy of these parameters across microbial targets, optimized protocols can be developed.

## RESULTS AND DISCUSSION

### Coculturing had no effect on surface attachment and cell removal.

Reduction of pathogenic bacteria and spoilage fungi on food manufacturing environmental surfaces represent two distinct goals of sanitation. Bacterial and fungal targets respond differently to various conditions in environmental sanitation, but a given sanitation treatment used by a commercial food facility must address both to ensure food safety and microbial quality (25). Moreover, microbial surface communities in food processing environments potentially include diverse populations. Previous work characterizing microbial cooccurrence on environmental surfaces has often used broad-stroke sampling methods such as swabbing (26–28). The implication that two isolates taken from a swab of multiple square inches does not necessarily indicate community interaction on more sparsely populated microbial surfaces but does suggest that a diversity of spoilage and pathogenic bacteria and fungi may be present. Assessing the impact of sanitation treatments on various microbial targets, in addition to the evaluation of the mediating impact of microbial targets on one another, remains an important consideration in the optimization of sanitation programs.

In this study, we selected two microbial targets, the foodborne pathogen L. monocytogenes and the food spoilage fungus, *Exophiala* spp. (colloquially referred to as “black yeast”), which have both been isolated from similar processing equipment niches in food plants (29–31). We initially sought to characterize the potential interactions between these two microbial targets during coupon attachment and in response to sanitation treatments as both cross-protecting and cross-sensitizing relationships have been observed between microbial community members (32–36). A full factorial study was designed across sanitation parameter treatment levels that totaled 288 possible combinations. Initially, 48 representative levels of treatment conditions were applied to (i) L. monocytogenes monocultured coupons, (ii) *Exophiala* spp. monocultured coupons, and (iii) L. monocytogenes and *Exophiala* spp. cocultured coupons to determine what, if any, effect coculturing had on attachment and removal of both microbial targets.

**FIG 1:**
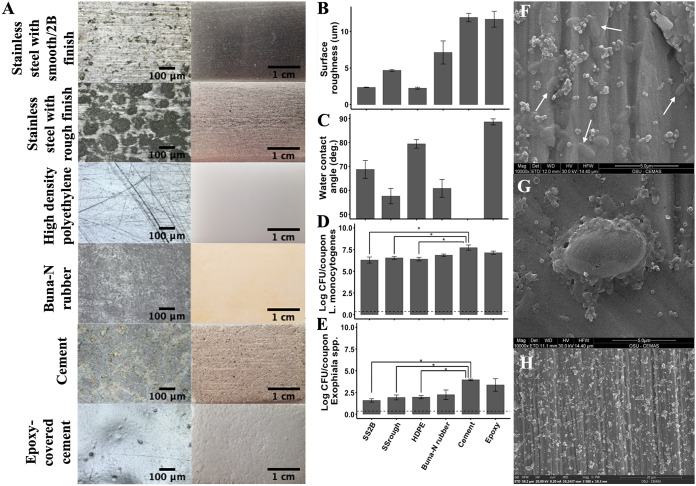
Surface material characterization and corresponding cell attachment. Surface material, laser micrographs (A, left), and images (A, right), (B) roughness (μm), and (C) hydrophobicity (degree). Initial counts of Listeria monocytogenes (D) and *Exophiala* spp. (E). Scanning electron microscopy images of a L. monocytogenes monoculture (F), an *Exophiala* spp. monoculture (G), and coculture on stainless steel coupon with a smooth/2B finish (H). Magnifications: ×10,000 (F and G), ×3,500 (H). The limit of detection is at 0.35 log CFU/coupon (dashed line). *, *P* < 0.05.

The 48 selected sanitation treatments included six types of surface materials, two levels of sodium hypochlorite-based sanitizer concentration, two levels of impeller-driven rotational velocity of the sanitizing fluid, two levels of contact time, and two levels of water temperature (see Fig. S1 and S2 in the supplemental material). Sanitation efficacy was compared between monocultured and cocultured coupons using analysis of variance (ANOVA) and a Tukey test. Coculturing did not significantly affect the initial cell attachment prior to treatment (*P* = 0.39 for L. monocytogenes counts and *P* = 0.15 for *Exophiala* spp. counts) (Fig. 1D and E). In addition, coculturing did not significantly change sanitation outcomes. The level of microbial removal was not significantly different for treated monocultured or cocultured coupons (*P* = 0.06 [see Table S1]; representative comparisons are shown in Fig. S1 and S2). Therefore, coculture inoculation procedures were applied in the remaining sanitation treatment experiments (see Fig. S3).

Coculturing has been shown to have heterogeneous effects on both community formation and sanitizer tolerance, generally explained by the difference between competitive and cooperative interactions among microorganisms. Govaert et al. (33) compared L. monocytogenes and *Salmonella* Typhimurium in both mono- and cocultured biofilms, finding that the coculture was less resistant to cold atmospheric plasma compared to monocultures, possibly due to the production of bacteriocins within the community. In contrast, Pang et al. (35) observed the cooperative interactions within the cocultured community. The additional EPS from Pseudomonas aeruginosa provided multilayer structure and extra protection against disinfection for *Salmonella* in coculture, in comparison to the scattered single cells or microclusters observed in *Salmonella* monoculture on coupons (35). Oxaran et al. (27) interchanged L. monocytogenes and Staphylococcus aureus with each of their associated environmental spoilage microbiota communities and concluded that enhanced protection is caused by associations in the biofilm instead of specific characteristics of the pathogen. In addition, the decreased diffusion of antimicrobial agents through multilayer complex structures has been shown to contribute to the increased resistance (32, 34, 37). A dense structure of multiple heterogeneous layers of L. monocytogenes and Lactobacillus plantarum cells increased sanitizer tolerance of both species against benzalkonium chloride, but the protective effect was less pronounced against peracetic acid (38). Gkana et al. (32) tested sublethal chemical disinfection on *S*. Typhimurium and S. aureus but did not observe a significant difference between monoculture and coculture inoculum preparation. Kostaki et al. (20) concluded that tolerance to treatments is independent of culture conditions and the observed effect depended both on the species used and the type of sanitizer applied. Similarly, coculturing did not significantly change the tolerance of either cell type to sanitation in this study which may be explained by the scattered distribution of single cells or micro clusters observed here as opposed to the complex multilayer biofilms associated with P. aeruginosa colonized by L. monocytogenes identified in other work (Fig. 1H) (35).

In addition to the lack of significant changes to the tolerance of microbiota to sanitation, neither did coculturing affect the attachment levels or distribution of the microbial targets on coupon surfaces (Fig. 1F to H). Visvalingam et al. (28) reported that antagonism was more prominent when the species in coculture require similar nutrients. In addition, species with a faster initial attachment during biofilm development or with a shorter generation time would dominate the other species (28, 39). Kostaki et al. (20) observed that antagonistic interactions were strain dependent and are marked by surface blanketing by one species and production of secondary metabolites as a hallmark of cross-kingdom antibiofilm behaviors (40). The attachment of L. monocytogenes and *Exophiala* spp. in this study were not spatial or interactive. Additional work comparing interactions among diverse fungal and bacterial communities is needed to fully characterize the mediating impact of coculturing on cleaning and sanitation outcomes.

### Only large differences in surface roughness facilitated the attachment of significantly higher levels of both *L. monocytogenes* and *Exophiala* spp.

Environmental surfaces have unique properties, such as surface topography, surface hydrophobicity, and physiochemical factors in the near-surface environment which can impact cell attachment and removal (41). The microscopic and macroscopic views of coupon surface materials are shown in Fig. 1A. Based on the macroscopic images, SS_rough_, cement, and epoxy coupons had uneven and porous surfaces (surface types and terms are explained in detail under “Coupon characterization” in Materials and Methods). Even though SS_2B_, high-density polyethylene (HDPE), and Buna-N rubber coupons seemed to have a homogenous surface topography, a substantial number of microvoids and grooves were present in the corresponding laser micrographs. Cell attachment to an inert surface results from complex physicochemical interactions among the cell, the surface, and the liquid phase (18). Roughness and hydrophobicity of the industrially relevant materials used in this study varied across different coupon surfaces, complicating the determination of clearly defined relationship among topology, hydrophobicity, and roughness among different surfaces often present in food manufacturing (Fig. 1B and C). However, generally, rougher surfaces facilitated the attachment of higher levels of both L. monocytogenes and *Exophiala* spp.

The cell envelope of L. monocytogenes and *Exophiala* spp. bear relatively nonpolar structures and attach more readily to hydrophobic surfaces (18, 42–44). Hydrophobic material surfaces produce water contact angles that approach and even exceed 90° due to characteristic water repelling (45, 46). Based on the contact angle goniometry of the surface materials in Fig. 1, hydrophobicity increased from SS_rough_ = 57.59° ± 9.66°; Buna-N rubber = 60.82° ± 11.13°; SS_2B_ = 68.75° ± 11.14°; HDPE = 79.42° ± 5.36°; to epoxy = 88.61° ± 3.78°, while the water contact angle for cement could not be determined due to surface porosity. The low surface tension of water on unsealed cement aids the cell penetration of the air-liquid interphase (41). However, there was not a clear relationship between hydrophobicity and microbial attachment (Fig. 1C to E) given the relative similarity of the goniometry among surfaces and the correlated variable of surface roughness.

The degree of attachment observed for L. monocytogenes and *Exophiala* spp. were similar across all surfaces. In general, larger surface roughness measurements are associated with increased cell attachment (Fig. 1D and E). The initial counts on SS_2B_ were 6.29 ± 0.60 log CFU L. monocytogenes/coupon and 1.58 ± 0.35 log CFU *Exophiala* spp./coupon. The initial counts on SS_rough_ were 6.54 ± 0.29 log CFU L. monocytogenes/coupon and 1.93 ± 0.49 log CFU *Exophiala* spp./coupon. The initial counts of L. monocytogenes were 6.40 ± 0.36 log CFU/coupon and 6.85 ± 0.23 log CFU/coupon on HDPE and rubber. The initial counts for *Exophiala* spp. were 1.96 ± 0.31 log CFU/coupon and 2.24 ± 0.96 log CFU/coupon on HDPE and rubber, respectively. Cement had the highest initial cell attachment with 7.72 ± 0.53 log CFU L. monocytogenes/coupon and 3.96 ± 0.16 log CFU *Exophiala* spp./coupon. The initial counts on epoxy were 7.13 ± 0.32 log CFU L. monocytogenes/coupon and 3.36 ± 1.24 log CFU *Exophiala* spp./coupon (Fig. 1D and E). Arithmetical average heights (R_a_) were used to characterize surface roughness. SS_2B_, SS_rough_, and HDPE had surface roughness, with average peak and trough differences of no more than 5 μm (2.3 ± 0.2 μm, 4.7 ± 0.5 μm, and 2.2 ± 0.5 μm, respectively). Buna-N rubber, cement and epoxy had higher surface roughness, 7.1 ± 6.1 μm, 11.9 ± 2.3 μm, and 11.7 ± 5.7 μm, respectively. Roughness is commonly used in the biofouling literature to characterize surfaces, but it does not account for spatial and hybrid parameters (47–51). Surface material roughness was a relevant variable for cell attachment in this study. The initial counts of attached L. monocytogenes cells on cement was significantly higher than those on SS_2B_ (*P* = 0.01), SS_rough_ (*P* = 0.04), and HDPE (*P* = 0.02) (Fig. 1D). Similarly, the level of attached cells of *Exophiala* spp. on cement was significantly greater than those on SS_2B_ (*P* = 0.01), SS_rough_ (*P* = 0.04), and HDPE (*P* = 0.04) (Fig. 1E). In contrast, there was not a significant difference in cell attachment among cement, Buna-N rubber, and epoxy for either L. monocytogenes or *Exophiala* spp. The initial counts on SS_2B_, SS_rough_, HDPE, Buna-N rubber, and epoxy were not significantly different from one another (*P* > 0.05).

Across these surfaces, sporadic, single-layered microcluster attachment of the microbial community was observed. Sessile cell distribution of both monoculture and coculture were examined after a 48-h incubation period by SEM (Fig. 1F to H). L. monocytogenes cells have been reported to be 0.4–0.5 μm in diameter by 1 to 2 μm long (52). Surface-adhered L. monocytogenes was determined to be 1.3 ± 0.2 μm by 0.6 ± 0.07 μm (Fig. 1F, arrows) and modest extracellular polysaccharide (EPS) secretion was evident, as described previously (53–56). Surface adhered *Exophiala* spp. were determined to be 6.6 ± 0.03 μm by 3.7 ± 0.03 μm, and greater EPS was observed (Fig. 1G). Yeast cells are the predominant morphotype of *Exophiala* spp. (57), and the mature single yeast cell swells to 5 to 6 μm by 4 μm for *E. phaeomuriformis* (*Sarcinomyces phaeomuriformis*) (58), while the mature single yeast cell of *E. dermatitidis* (*Wangiella dermatitidis*) is ∼7 μm by 5 μm (59). The cocultured surfaces similarly had single-layered cells and microclusters sporadically distributed as opposed to a complex honeycomb-like or fimbria-like structure associated with true biofilm communities. Bremer et al. (17) observed similar L. monocytogenes single cells or monolayer communities on conveyor belt (PVC-Nonex) material. Park and Kang (6) observed L. monocytogenes single-cell aggregation in cracks and crevices of produce or food contact surfaces. Abdo et al. (60) observed similar microcolonies of Saccharomyces cerevisiae on stainless steel chips. Figure 1H shows a uniform distribution of L. monocytogenes and *Exophiala* spp. cells with limited interaction in the *z* axis, which supports the finding that coculturing these organisms did not result in significant differences in surface colonization or tolerance to removal.

### Application of CFD simulations provided estimations of wall shear stress in a bench-scale bioreactor system that mimicked environmental sanitation.

Environmental sanitation employs both mechanical and chemical forces to overcome the bonds between cells and material surfaces, rendering shear stress an essential factor to include in simulation studies (61). However, shear stress is frequently neglected from *in vitro* evaluations of environmental sanitation on coupon surfaces (3–7). While this work provides insight into the contribution of sanitizer chemistry, local wall shear stress has been identified as a driving force in deposit and biofilm removal (62). Previous studies have found that applying sanitizer quiescently did not remove laboratory or industrial biofilms without incorporating shear stress (3, 16, 63). Commercially, detergents and sanitizers are paired with mechanical action to effectively remove food residues and microbiota, but incorporation of reproducible shear stress in bench-scale simulations of environmental sanitation activities remains a challenge (3). In this study, shear stress was quantified through the use of CFD applied to a stirred vessel bioreactor.

**FIG 2:**
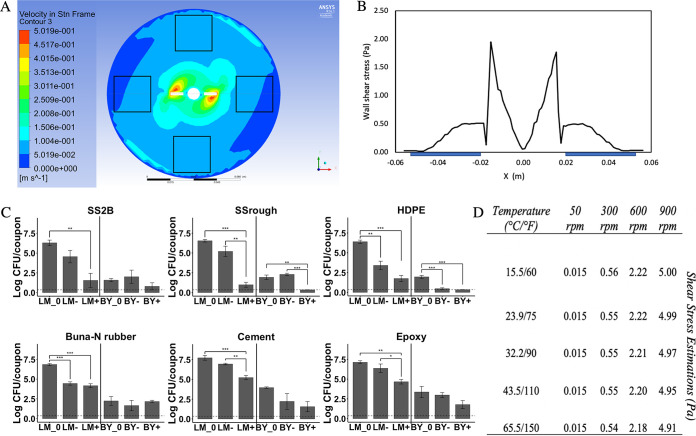
Evaluation of bench-scale sanitation bioreactor treatments. (A) Velocity distribution on the bottom of the vessel at the steady state. Black squares indicate the location of the coupons. White blocks indicate the location of the impeller (obtained from Fan et al. [64]). (B) The wall shear stress distribution along the center line on the bottom plane of the stirring beaker, and the blue bands indicate the location of the sample coupons (obtained from Fan et al. [64]). (C) Absolute survivor counts under the most and least intense treatment levels. Comparison of untreated control coupons (LM_0 and BY_0) and survivors following the least intense (LM– and BY–) treatment combination (0.6 ml/liter sanitizer at 50 rpm for 30 s at 23.9°C) and survivors following the most intense (LM+ and BY+) treatment combination (2.4 ml/liter sanitizer at 900 rpm for 5 min at 23.9°C). LM, L. monocytogenes survivor counts; BY, black yeast (i.e., *Exophiala* spp.) survivor counts. *, *P* < 0.05; **, *P* < 0.01; ***, *P* < 0.001. The limit of detection is at 0.35 log CFU/coupon (dashed line). (D) Estimated wall shear stress magnitude (Pa) of the stirred vessel from CFD simulations under various temperature and impeller rotational velocity combinations.

CFD simulations resulted in predicted velocity distribution in the horizontal plane of the vessel bottom where coupons were located (Fig. 2A) (64). The fluid velocity and flow gradients were both greatest near the impeller region where velocity contours were most dense. In contrast, the flow had a lower velocity and smaller gradient outside the immediate impeller region, and no flow split or backward portion was identified. The impeller tip where momentum input was greatest resulted in two peaks in the shear stress distribution (Fig. 2B) (64). Consequently, coupons were located outside the immediate impeller region. The reported shear stress exerted by the sanitizing fluid on the coupon surface reflects an average across all cells included in the coupon sites. The estimated mean shear stress ranged from 0.015 to 5.00 Pa when the rotational speed of the impeller was between 50 and 900 rpm at 15.6 to 32.2°C. The magnitude of the shear stress increased as rotational speeds increased. In addition, while the temperature of the fluid impacts viscosity and the derived shear stress, changes in temperature under the modest ranges evaluated here had minimal impact on local wall shear stress when compared to the impact of the rotational speed of the impeller (Fig. 2D).

The impact of shear stress in environmental sanitation is analogous to CIP treatments of bulk tanks where spray devices suspended in the center of the tank direct water sprays on tank surfaces (65). The impinging water or sanitizer streams are designed to remove soil or microbial targets through a combination of factors, including shear at the point of spray impingement and shear from the film of fluid falling down the tank surface. The shear from falling fluid is much lower but represents the treatment applied to the majority of the tank’s surface area. Direct spray is often applied to the upper third of the tank surface and the falling liquid film irrigates the remaining surface (66). A Reynolds number of at least 2,000 is required in the irrigating fluid film to remove soils (66). The Reynolds number is the ratio of inertial stress to viscous stress (i.e., shear stress) used to categorize fluid systems (67). The bench-scale bioreactor obtained a Reynolds number of 5,000 when the shear stress was at 0.015 Pa, which supports the validity of this bench-scale bioreactor as a model of environmental sanitation. However, direct impingement of high velocity water or from sanitizer hoses, especially when held close to environmental surfaces, would likely represent greater shear stress than that achieved within the bioreactor at the highest impeller rotational velocity. Though similar to bulk tank sanitation, these treatments may represent the minority of shear stress exposures across the total surface area in manufacturing space.

In addition to controlling shear stress, the bench-scale sanitation bioreactor facilitated parameterization of a range of sanitation treatment variables. This allowed for evaluation of the interactions among complex variables on the reduction of surface microbiota. The microbial reductions achieved under the most intense (2.4 ml/liter sanitizer, 4.99 Pa shear stress, and 5 min) and least intense (0.6 ml/liter sanitizer, 0.015 Pa shear stress, and 30 s) treatments at 23.9°C (75°F) achieved within this model system are shown in Fig. 2C. The impact of surface material on microbial reduction during sanitation was even more pronounced than the impact of surface material on initial cell attachment (Fig. 2C; Fig. 1D and E). Significant reductions of L. monocytogenes were achieved under the most intense sanitation treatments across all surface material types; however, the absolute level of surviving L. monocytogenes varied based on surface material (Fig. 2C). Surface materials that had roughnesses of <5 μm (SS_2B_, SS_rough_, and HDPE) retained survivors of 1.5 ± 1.5 log CFU/coupon, 1.0 ± 0.5 log CFU/coupon, and 1.8 ± 0.7 log CFU/coupon of L. monocytogenes, respectively, following the most intense treatments. Meanwhile, the levels of L. monocytogenes survivors were 4.2 ± 0.4 log CFU/coupon, 5.2 ± 0.5 log CFU/coupon, and 4.7 ± 0.5 log CFU/coupon on Buna-N rubber, cement, and epoxy. *Exophiala* sp. counts were reduced below the limit of detection on SS_rough_ and HDPE following the most intense treatment. However, no significant reduction was observed on the remaining coupons. The level of *Exophiala* spp. survivors were 1.38 ± 0.80 log CFU/coupon on SS_2B_, 2.77 ± 0.27 log CFU/coupon on Buna-N rubber, 2.13 ± 1.13 log CFU/coupon on cement, and 2.38 ± 0.88 log CFU/coupon on epoxy.

In comparison, the least intense treatment level did not yield a statistically significant reduction of L. monocytogenes and *Exophiala* spp. across almost all surface materials (*P* > 0.05). Even though the reduction on HDPE and Buna-N rubber were significant (*P* = 0.004 and *P* = 0.0004), a relatively high number of L. monocytogenes cells survived the treatment (3.4 ± 0.9 and 4.4 ± 0.4 log CFU/coupon). In previous studies that applied mechanical and chemical treatments simultaneously, the impact of increased sanitizer concentration (0 to 500 mg/liter sodium hypochlorite) did not significantly reduce P. fluorescens on stainless steel cylinders, while an increase in the Reynolds number (4,000 to 161,000) facilitated significant biofilm detachment (68). However, the study monitored percentage of biofilm loss as a function of increase in the Reynolds number, which limited comparisons between individual mechanical treatment parameters. Gião and Keevil (19) discovered that surface-adhered L. monocytogenes visualized by microscopy was reduced by 98% of the surface area coverage under a shear stress of 24 to 144 Pa without chemical treatment on stainless steel surfaces, while a more hydrophobic material, polytetrafluoroethylene, had significantly less cell removal by shear stress. In some studies, agitation has been incorporated using an orbital shaker (69, 70). Goode et al. (71) studied the effects of chemical concentration, flow velocity, and temperature on sanitation and found that a NaOH-based cleaning agent in removing yeast from industrial stainless-steel surfaces at flow velocities of 0.26 to 0.5 m s^−1^. However, it is impossible to translate laboratory sanitation parameters to outcomes in a commercial setting without designated mathematic tools such as CFD.

### The relative importance of sanitation parameters differs based on microbial target.

**FIG 3:**
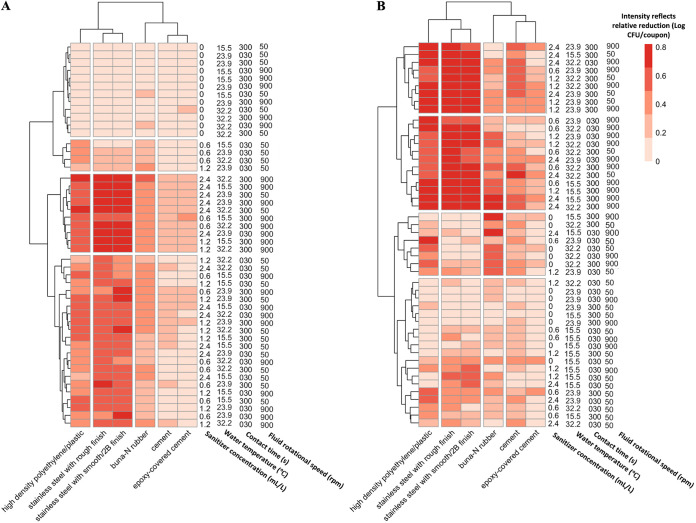
Relative reduction (Δ*N*/*N*_0_ ratio) of L. monocytogenes (A) and *Exophiala* spp. (B) after sanitation. Columns represent surface material; rows represent treatment combinations.

As sanitation treatment parameters were varied, the relative reductions achieved under these treatments differed between bacteria and fungi, which are not typically assessed in combination despite the fact that industrial sanitation programs must address both (Fig. 3). The relative reduction ranged from 0.0 to 0.82 (ratio in log numbers) for *Exophiala* spp., which corresponded to a 0.0 to 2.21 log CFU/coupon reduction, and the relative reduction ranged from 0.0 to 0.93 in L. monocytogenes which corresponded to a 0.0 to 6.19 log CFU/coupon reduction (Fig. 3). According to the U.S. Environmental Protection Agency (EPA) Sanitizer Product Performance Test Guidelines for use on food contact and non-food contact surfaces, sanitizers for non-food contact surfaces must achieve a 3-log CFU/ml reduction in 5 min against S. aureus, Klebsiella pneumoniae, and Enterobacter aerogenes (72). Nonhalide sanitizers for food contact surfaces must achieve a 5-log CFU/ml reduction in 30 s when applied to Escherichia coli and S. aureus. However, these standards apply to planktonic cell treatments that neglect surface effects and shear stress. Based on our data, the relative reduction of L. monocytogenes never exceeded 0.38 on cement and epoxy under 30 s of treatment. Buna-N rubber achieved 0.22 to 0.65 relative reduction under 30 s when sanitizer was used. The use of sanitizer successfully reduced cell counts on SS_2B_, SS_rough_, and HDPE no matter what shear stress was applied, leading to relative reduction of 0.15 to 0.88, 0.17 to 0.93, and 0.27 to 0.85, respectively.

The absolute log reductions achieved against *Exophiala* spp. are difficult to directly compared to those for L. monocytogenes since the initial counts were much lower. However, relative reductions in L. monocytogenes and *Exophiala* spp. responded differently to various sanitation treatment combinations. Treatment conditions were grouped based on relative reduction for either L. monocytogenes or *Exophiala* spp. using a cluster analysis shown in Fig. 3. While direct extrapolation of bench scale log reduction findings to commercial outcomes is difficult, the application of hierarchical clustering facilitates grouping of diverse treatment combinations which yield similar relative reductions allowing for the determination of compensatory variables (see Fig. S5). For example, every treatment combination applied to L. monocytogenes which included 0.0 ml/liter sanitizer concentration was clustered in one group due to the limited relative reduction achieved (Fig. 3A). Meanwhile, the treatment combinations applied to *Exophiala* spp. which included a 0.0 ml/liter sanitizer concentration were variably grouped with higher concentration levels paired with either lower fluid velocity (which corresponds directly with shear stress, Fig. 2D) or short contact time.

All sanitation parameters impacted the reduction of surface microbiota. The results of the ANOVA from five-way interaction models showed that surface material, sanitizer concentration, contact time, and impeller-driven fluid rotational velocity significantly affected the relative reduction of L. monocytogenes (*P* < 0.0001; Table 1). Water temperature was not a significant variable in L. monocytogenes control within the range evaluated in this study (15.5 to 32.2°C). In contrast, studies on CIP operations reported that increasing water temperature (up to 50°C) enhanced sanitation outcomes compared to room temperature water (73–75). Fan et al. (76) determined that increasing water temperature (from 23 to 45°C) also improved the effectiveness of removing a protein-based cohesive solid foulant in commercial pipes. High water temperatures are often applied in CIP operations to increase the efficacy of sanitizer. However, a water temperature of >35°C is less commonly used in environmental sanitation and the modest temperatures ranges applied therein may not be large enough to significantly impact L. monocytogenes reduction. In contrast, temperature was considered a significant factor in *Exophiala* spp. reduction (Table 2). A potential explanation for this may be a consequence of the increased EPS production associated with microbial surface specialists, such as black yeast (29), as observed in Fig. 1G. The polysaccharide component of EPS is a target for sanitizer oxidation. An increase in ambient temperature may drive the generation of the active hypochlorite species which can then readily degrade the EPS and render *Exophiala* spp. cells more susceptible to removal. Similarly, increased EPS degradation could also increase exposure of the cells to sanitizer. Removal (via sheer stress) and inactivation (via sanitizer exposure) were not differentiated by enumeration. Therefore, the effect of elevated treatment temperature on EPS degradation may enhance the reduction of *Exophiala* spp. through both mechanisms.

**TABLE 1:** ANOVA results based on microbial relative reduction to partition source of variation (material, sanitizer concentration, water temperature, contact time, fluid rotational speed, and interactions) for L. monocytogenes in the coculture model^*a*^

Source of variation or factor	*df*	Mean squares	*F* ratio	*P*
Material	5	6.390	493.100	<0.0001
Sanitizer concn	1	1.469	113.325	<0.0001
Contact time	1	2.885	222.591	<0.0001
Fluid rotational speed	1	1.113	85.850	<0.0001
Material × sanitizer concn	5	0.062	4.774	0.0003
Material × contact time	5	0.073	5.646	<0.0001
Sanitizer concn × contact time	1	0.0001	0.005	0.944
Material × fluid rotational speed	5	0.047	3.589	0.003
Sanitizer concn × fluid rotational speed	1	0.026	1.993	0.159
Contact time × fluid rotational speed	1	0.110	8.520	0.003
Material × sanitizer concn × contact time	5	0.031	2.376	0.038
Material × sanitizer concn × fluid rotational speed	5	0.030	2.307	0.043
Material × contact time × fluid rotational speed	5	0.059	4.584	0.0004
Sanitizer concn × contact time × fluid rotational speed	1	0.004	0.280	0.597
Material × sanitizer concn × contact time × fluid rotational speed	5	0.030	2.315	0.042
Residuals	600	0.013		

a Insignificant variables were excluded by the model-simplifying function.

**TABLE 2:** ANOVA results based on microbial relative reduction to partition source of variation (material, sanitizer concentration, water temperature, contact time, fluid rotational speed, and interactions) for the *Exophiala* spp. in the coculture model^*a*^

Source of variation or factor	*df*	Mean squares	*F* ratio	*P*
Material	5	1.222	19.338	<0.0001
Sanitizer concn	1	0.817	12.932	0.0004
Water temp	1	0.909	14.389	0.0002
Contact time	1	5.941	94.056	<0.0001
Fluid rotational speed	1	4.599	72.813	<0.0001
Material × sanitizer concn	5	0.056	0.892	0.486
Material × water temp	5	0.019	0.306	0.909
Material × contact time	5	0.207	3.282	0.006
Sanitizer concn × contact time	1	0.068	1.079	0.299
Water temp × contact time	1	0.121	1.919	0.167
Material × fluid rotational speed	5	0.117	1.856	0.100
Sanitizer concn × fluid rotational speed	1	0.021	0.329	0.566
Water temp × fluid rotational speed	1	0.095	1.503	0.221
Contact time × fluid rotational speed	1	0.081	1.286	0.257
Material × water temp × contact time	5	0.054	0.861	0.507
Material × sanitizer concn × fluid rotational speed	5	0.141	2.225	0.050
Material × water temp × fluid rotational speed	5	0.184	2.909	0.013
Material × contact time × fluid rotational speed	5	0.088	1.399	0.223
Sanitizer concn × contact time × fluid rotational speed	1	0.237	3.748	0.053
Water temp × contact time × fluid rotational speed	1	3.129	49.539	<0.0001
Material × water temp × contact time × fluid rotational speed	5	0.168	2.664	0.022
Residuals	586	0.063		

a Insignificant variables were excluded by the model-simplifying function.

**FIG 4:**
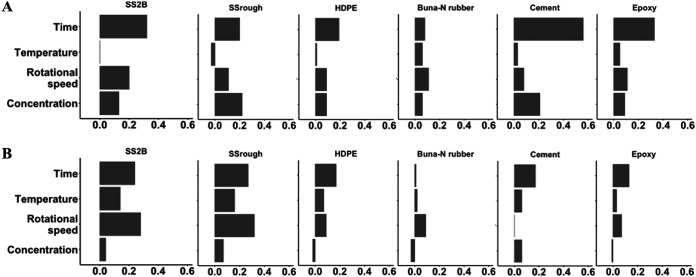
Change in adjusted *R*^2^ value when the variable was last added to the four-way interaction L. monocytogenes (A) and *Exophiala* sp. (B) models on various surfaces.

While most parameters were considered statistically significant against both L. monocytogenes or *Exophiala* spp., the relative contribution of each variable varied by microbial target and was evaluated using the predicted change in *R*^2^ for each statistical model across surface material (Fig. 4). Treatments that included longer contact times were clustered together based on higher relative reductions in the L. monocytogenes model (Fig. 3A). The change in *R*^2^ associated with the variable of contact time also ranked as the most important predictor across most surface materials, except for SS_rough_ and Buna-N rubber (Fig. 4A). For *Exophiala* spp., treatment combinations with longer contact times and higher fluid rotational velocity were clustered together and resulted in greater relative reductions (Fig. 3B). Given that impeller driven fluid velocity directly determined wall shear stress (Fig. 2D), shear stress appeared to be the most important predictive factor on SS_2B_, SS_rough_, and Buna-N rubber (Fig. 4B). Contact time remained the most important predictive factor on HDPE, cement, and epoxy (Fig. 4B). The comparison of clusters of treatment combinations based on outcomes may facilitate the development of sanitation regimes where longer contact time or greater shear stress are applied so that lower sanitizer concentrations can be used. The Sinner’s Circle was previously developed to describe the relationship among four key factors that determine the success of industrial cleaning processes: chemistry, temperature, contact time, and mechanical power. Consequently, the compensatory effect within the Sinner’s Circle has been investigated in laundry and textile hygiene studies which illustrated that a decrease in one portion of the sanitation program could be partially compensated for by increasing one or more of the other factors (77–81). However, application of modified programs would still require pilot-scale testing and close initial monitoring to ensure desired sanitation outcomes.

Rotational fluid velocity (e.g., shear stress) contributed the most to *Exophiala* spp. removal on SS2B, SS_rough_, and Buna-N rubber (Fig. 4). In contrast, it only represented the most explanatory variable in the L. monocytogenes model for Buna-N rubber. Similarly, the relationship between shear stress and surface material features may explain the significant differences in L. monocytogenes survivorship following the most and least intense treatment levels on SS_rough_, cement, and epoxy, but not on SS_2B_, HDPE, and Buna-N rubber (Fig. 2C). The niches between peaks and troughs on a surface can harbor microbial colonizers by reducing the effective shear stress during sanitation (82). However, the relative size and shape of the cells to those surface features also play a role in adherence (41, 83). The peak and trough differences on SS2B, SS_rough_, and HDPE were smaller than the *Exophiala* spp. cell size (6.6 ± 0.03 μm by 3.7 ± 0.03 μm). While the surface roughness measurements on Buna-N rubber were similar to the approximate length of black yeast cells, the surface roughness measurements for cement and epoxy exceeded the approximate cell size of black yeast. All surface roughness measurements exceeded the approximate L. monocytogenes cell size (1.3 ± 0.2 μm by 0.6 ± 0.07 μm) which may account for differences in the importance of shear stress variation between the two microbial targets. Whitehead et al. (84) observed the ease of removal of P. aeruginosa cells (1 to 3 μm) from 0.5-μm featured surfaces. In this study, shear stress was not as effective on HDPE, cement, and epoxy. The high surface roughness of cement and epoxy offered protection against high shear stress during sanitation. HDPE is a relatively hydrophobic surface compared to other materials in the same range of roughness levels. In addition, the laser micrograph showed numerous grooves on HDPE, even while the average surface roughness of HDPE was low (Fig. 1A and B). Katsikogianni and Missirlis (82) claimed that R_a_ or R_q_ values cannot represent defects or crevices in the surface finish with great sensitivity because they are means taken over a certain path length. Park and Kang (6) claimed that the existence of crevices was more important than the R_a_ and R_q_ values in the inactivation patterns of pathogens, even at the microscopic level.

### Conclusions.

Our assessment of the impact of surface sanitation treatments on both L. monocytogenes and the fungal spoilage functional group, black yeast, provided insights into sanitation efficacy across microbial kingdoms. However, the use of three species (three L. monocytogenes isolates, *E. phaeomuriformis*, and *E. dermatitidis*) provides only high-level insights regarding the difference between fungal and bacterial targets, leaving many remaining comparisons among other individual bacterial and fungal species. At this high level, one notable difference between bacterial foodborne pathogens and fungal spoilage microbes is the variation in typical cell sizes which may represent an important difference in ranking the relative contribution of sanitation program features between the two groups. This study also incorporated the impact of shear stress in environmental sanitation, as well as the interactions between surface material and microbial target within a bench-scale model. The results indicated that sanitation parameters impacted L. monocytogenes and *Exophiala* survival on food contact surfaces differently. For example, the size difference between fungal and bacterial cells mediated the effect of shear stress and material surface roughness. The increased EPS production by *Exophiala* spp. may have contributed to the increased role of temperature in enhancing black yeast removal compared to L. monocytogenes control. And, by comparison, chemical inactivation rather than mechanical removal appeared to be a primary feature in L. monocytogenes control. Our data show that many attributes (i.e., concentration-contact time or shear force-contact time) in sanitation are compensatory, suggesting that some features of a sanitation regimen may be lowered if others are concomitantly increased. Even though all sanitation parameters impact the efficacy of sanitation activities, evaluation of the relative importance of individual sanitation parameters will allow food processors to balance tradeoffs between quality and safety with the cost of implementation and waste stream management, as appropriate for their facility. The bench-scale system described in this study provides a foundation to bridge experimental data and commercial applications. The application of CFD in a bench-scale bioreactor system can further improve the quantitative evaluation of complex sanitation programs in the food manufacturing environment and reduce the burden of pilot-plant validation trails by providing an *in vitro* model for environmental sanitation.

## MATERIALS AND METHODS

### Strains and inoculum preparation.

Three strains of the bacterial pathogen L. monocytogenes—FSL R2-0574 (85), FSL F6-0665, and FSL M2-0018 (86)—were kindly provided by the Food Safety Lab in the Department of Food Science of Cornell University. The L. monocytogenes were isolated from cheese manufacturing facilities in New York and California. *Exophiala phaeomuriformis* E2-0572 was isolated from hot-filled fruit filling from a yogurt plant. *Exophiala dermatitidis* YB-734 was obtained from the USDA-ARS Culture Collection NRRL (Northern Regional Research Laboratory), and its isolation source was unknown. L. monocytogenes strains were grown separately in 5 ml of tryptic soy broth (TSB; Becton, Dickinson, and Co. [BD], Sparks, MD) at 30°C from cultures stored at −80°C in TSB containing 25% (vol/vol) glycerol. Fungal cultures were stored at −80°C in malt extract broth (MEB; BD) containing 25% (vol/vol) glycerol prior to use. *Exophiala* spp. frozen stock cultures were plated on malt extract agar (MEA; BD) and incubated at 25°C for 28 days prior to use in the experiment. Inocula were prepared by scraping plates and then washing and resuspending the cells in 0.1% buffered peptone water (BD).

### Coupon characterization.

Coupon materials included stainless steel with a smooth, 2B finish (referred to as SS_2B_); stainless steel with an unpolished finish (referred to as SS_rough_); high-density polyethylene (referred to as HDPE); Buna-N rubber (60A, plain backing type, 450% elongation, white); unsealed cement (referred to as cement); and epoxy-coated cement (referred to as epoxy). Unsealed cement coupons were fabricated manually by mixing 675.29 g of Quikrete anchoring cement (10 lb, purchased from a national retailer; Quikrete, Ithaca, NY) with 877.5 g of water. The cement and water mixture was then molded into the size of the designed coupon (2.4 cm in width, 3.5 cm in length, and 0.48 cm in thickness). Half of the unsealed cement coupons were then coated with water-based epoxy coating (Rust-Oleum epoxy shield 2-part gray gloss garage floor epoxy). The characterization of surface materials was performed prior to surface inoculation. Surface roughness was measured using laser microscopy (VK-X200 series; Keyence, Osaka, Japan) with a 50× lens objective and analyzed with VK Viewer software (Keyence, Osaka, Japan). Surface hydrophobicity was measured using a contact angle instrument (Rame-Hart Instrument Co., Succasunna, NJ) paired with DROPimage Advanced software (Rame-Hart) under 23°C and 28% relative humidity.

### Surface-adhered cell attachment.

Coinoculated coupons were inoculated with a suspension of the two black-yeast (BY) *Exophiala* spp. and the three L. monocytogenes strains. The monocultured coupons were inoculated with either the three L. monocytogenes strains (referred to as LM monoculture) or the two *Exophiala* strains (referred to as BY monoculture). A static culturing method was utilized in generating surface-adhered cells to reduce variability and better replicate quiescent fluids on environmental surfaces. Sterile coupons were placed vertically in a sterile 50-ml beaker containing 20 ml of inoculum (7.2 log CFU/ml L. monocytogenes and 7.0 log CFU/ml *Exophiala* spp. in TSB) to reduce settling of nonadhered cells onto the surface. The reaction chamber was incubated at 25°C for 24 h. Subsequently, the coupon was gently transferred to uninoculated TSB at 25°C for another 24 h to allow the production of a more mature biofilm (4, 27, 87, 88). To assess cell accumulation and reproducibility, coupons were sampled at 24-h intervals throughout a 96-h incubation period. Coupons were rinsed with 6 ml of buffered peptone water (0.1%) and plated on MOX and MEA for enumeration. Surface-adhered cell counts did not change significantly after 48 h of incubation (data not shown).

### Bench-scale sanitation system.

A bench-scale sanitation system (see Fig. S4A) was used in the treatment of surface-adhered cells on coupons. The cylindrical, jacketed, double-walled glass vessel (11.2 cm in diameter) was connected to a water bath and temperature-controlled water was pumped through the double walls. A double-turbine-baffled digital stirrer (2.8 cm in diameter) located at the center of the vessel provided controlled turbulence for the treatment (see Fig. S4B). The distance between impeller and the beaker bottom was 0.32 cm. A double turbine baffled stirrer was located at the center of the vessel to provide a range of fluid characteristics (see Fig. S4C and S4D). There were four symmetrically positioned coupons located at the bottom of the vessel which were anchored by a customized rubber sheet. The rubber sheet provided a smooth transition to the leading edge of each coupon and assisted in preventing an unwanted vortex near the coupon surface. Essential sanitation parameters, including the flow characteristic of the sanitizer solution, the temperature of the sanitizer solution, the concentration of the solution, and the contact time, were controllable using this system.

### CFD simulations and calculations of shear stress.

CFD simulations were adapted from Fan et al. (64). Briefly, ANSYS CFD (ANSYS, Inc., Canonsburg, PA) software was used for simulations in this study. Mesh creation was performed in ANSYS CFD software ICEM 15.0. A computational grid was created to describe the controlled volume occupied by the fluid inside the bioreactor. Velocity and wall shear stress were determined by the computational model and stored in the cells. The geometry of the jacketed vessel was created based on the actual dimension, location, and the shape of the impeller and the vessel (see Fig. S4B to D). The computational grid consisted of a total number of 4.5 × 10^5^ active cells in an unstructured, patch dependent, triangular prism mesh. Due to the complexity of the motion of impeller in the vessel, 40% of the total mesh cells were assigned to the impeller region (less than 10% of the total vessel volume) so as to resolve the steep velocity gradients in the impeller region. ANSYS Fluent 6.3 was used to obtain a set of discrete algebraic equations for flow variable simulation by using a control-volume approach and integrating the governing equations over each cell in the mesh. The flux of fluid through the cell faces was obtained by interpolation using different numerical techniques so that all the fluid variables were found at each cell node. A multiple rotating reference frame approach was implemented for impeller modeling (12). The flow followed the laminar model. The velocity magnitude contour of the vessel bottom was obtained from the convergence of the simulation in Fluent. The shear stress $\tau_{w}$ values on the soiled coupon were quantified with CFD-POST based on the following equation:

$$\tau_{w}=\mu {\left(\frac{\partial u}{\partial y}\right)}_{y=0}$$

where μ is the dynamic viscosity of the fluid (Pa⋅s), *y* is the distance from solid wall, and ∂*_u_*/∂*_y_* is the velocity magnitude contour.

### Treatment on surface-adhered cells.

Inoculated coupons were treated with 0.0, 0.6, 1.2, and 2.4 ml/liter sodium hypochlorite-based sanitizer (Ecolab, Inc., St. Paul, MN; active ingredient, 8.4% sodium hypochlorite) for either 30 s or 5 min at 15.5°C (60°F), 23.9°C (75°F), and 32.2°C (90°F) at 50- and 900-rpm impeller rotational velocities (see Fig. S3). The sanitizer concentrations represent the top (2.4 ml/liter) and bottom (1.2 ml/liter) of the manufacturer’s recommended dose, an additional half-fold dilution (0.6 ml/liter), and a control (0.0 ml/liter). At the end of each treatment, concentrated Dey-Engley neutralizing broth (BD) was added to the vessel to stop the sanitizer reaction. The bioreactor vessel was disinfected with 70% ethanol and rinsed three times with deionized water between each treatment. Coupons were disinfected between experiments with 6.15% sodium hypochlorite solution, rinsed for 30 s with running water, and autoclaved prior to use. Each experimental condition was performed in triplicate.

### Recovery and enumeration.

Coupons were rinsed with 6 ml buffered peptone solutions (0.1%) to remove nonadhered cells. Survivors were harvested by scraping the coupon surface using sterile wood applicators (Puritan Medical Products Company LLC, Guilford, ME), followed by agitation to break up aggregates per EPA standard methods (89). Recovered cells were then plated on MOX (modified Oxford agar) for L. monocytogenes enumeration and MEA for *Exophiala* spp. enumeration. Untreated control coupons were sampled to determine initial counts. MOX plates were incubated at 35°C for 48 h, followed by enumeration. Fungal colonies were enumerated after incubation at 25°C for 5 days. To quantify the effect of using the selective medium, MOX, to recover potentially injured L. monocytogenes cells, a subset of sanitizer treatments was applied to planktonic L. monocytogenes prior to replica plating on both TSA and MOX. Cells were treated with sanitizer concentrations of 0.3, 0.6, 1.2, and 2.4 ml/liter for 30 s and 300 s at 23.9°C (75°F) to assess the effect and degree of cell injury. Survivors were plated on both TSA and MOX and incubated at 35°C for 48 h, followed by enumeration. ANOVA and *post hoc* Tukey tests revealed no significant difference between survivor counts recovered from TSA and MOX (*P* = 0.97) and, in most instances, counts from MOX plates were slightly numerically greater.

### Statistical analysis.

All statistical analyses were performed in R (version 3.3.1; R studio, Boston, MA) (Table 2). In order to test for the effect of inoculation method (coculture, LM monoculture, and BY monoculture) on attachment and removal, a seven-way interaction model based on both L. monocytogenes and *Exophiala* spp. relative reduction from monoculture and coculture was analyzed. Data were analyzed using the following model: relative reduction (LM and BY) = surface material × sanitizer concentration × water temperature × intensity of washing × inoculum × cell type, where *P* < 0.05 was considered significant. Relative reduction was calculated using the following equation: relative reduction = (log *N*_0_ – log *N*)/log *N*_0_, where *N*_0_ is the initial population and *N* is the survivor count following treatment. Relative reduction, instead of absolute reduction, was used due to the variation in initial counts between L. monocytogenes (an average of 6.8 log CFU/coupon) and *Exophiala* spp. (an average of 2.5 log CFU/coupon). Backwise stepwise selection using stepAIC function from MASS package in R (90) was used to remove terms that did not improve model fit by two AIC units (91).

Initial counts of L. monocytogenes and *Exophiala* spp. were analyzed in separate linear regression models. Pairwise comparison between each surface material was evaluated using the least square means (emmeans) package in R (92). ANOVA and *post hoc* Tukey tests were performed to evaluate statistically significant differences between the absolute survivor counts of L. monocytogenes or *Exophiala* spp. under the most intense and least intense treatment combination. No significant difference was observed between survivor counts from untreated and water-treated coupons (0.0 ml/liter sanitizer), and therefore, every treatment combination paired with 0.0 ml/liter was not included in the model in order to avoid amplifying the significance of change in sanitizer concentration in the model. The full-factorial coculture data were analyzed using the following five-way interaction model: relative reduction (LM or BY) = surface material × sanitizer concentration × water temperature × contact time × shear stress, where *P* < 0.05 was considered significant. Separate models were used for L. monocytogenes and *Exophiala* spp. Similarly, backwise stepwise selection using stepAIC function was used to simplify the model. ANOVA was performed on the data. The summary of stepAIC function was used to estimate the *R*^2^ value of the equation. The change in *R*^2^ that each fixed variable produces when added to a model that contains all of the other variables represents the percentage of the variance a given variable explains. It was used to rank the relative importance of each significant predictor variable from the ANOVA test.

## Supplementary Material

Supplemental file 1
