# Supplementary material for: All Treatment Parameters Affect Environmental Surface Sanitation Efficacy, but Their Relative Importance Depends on the Microbial Target
Source: Appl Environ Microbiol. 2020 Dec 17;87(1):e01748-20. doi: 10.1128/AEM.01748-20 (PMC7755260; doi:10.1128/AEM.01748-20)
Supplement: Supplemental file 1 [file AEM.01748-20-s0001.pdf]

Table S1. ANOVA results based on microbial relative reduction to partition source of variation (material, sanitizer concentration, water temperature, intensity of washing, inoculum, cell type and interactions) for monoculture and co-culture combined model. Insignificant variables were excluded by the model simplifying function.

Source of variation or factor	*df*	Mean Square	*F* ratio	*P*-value
Material	5	2.4011	50.9445	< 0.0001
Sanitizer concentration	1	1.0302	21.8577	< 0.0001
Water temperature	1	0.0003	0.0057	0.940
Fluid rotational speed	1	9.1283	193.6811	< 0.0001
Inoculum (mono vs. poly)	1	0.1641	3.4811	0.063
Cell type	1	0.3743	7.9426	0.005
Material×Sanitizer concentration	5	0.1032	2.1891	0.054
Material×Fluid rotational speed	5	0.2342	4.9693	0.0001
Sanitizer concentration×Fluid rotational speed	1	0.0409	0.8686	0.5352
Water temperature×Fluid rotational speed	1	0.0040	0.0852	0.770
Material×Inoculum	5	0.1745	3.7025	0.003
Water temperature×Inoculum	1	0.0032	0.0672	0.796
Fluid rotational speed×Inoculum	1	0.0039	0.0826	0.774
Material×Cell type	5	0.6642	14.0917	<0.0001
Fluid rotational speed×Cell type	1	0.8603	18.2533	<0.0001
Inoculum×Cell type	1	0.0060	0.1275	0.721
Material×Sanitizer Concentration×Fluid rotational speed	5	0.2250	4.7740	0.0003
Water temperature×Fluid rotational speed×Inoculum	1	0.2182	4.6287	0.032
Material×Inoculum×Cell type	5	0.3226	6.8441	<0.0001
Residuals	528	0.0471		

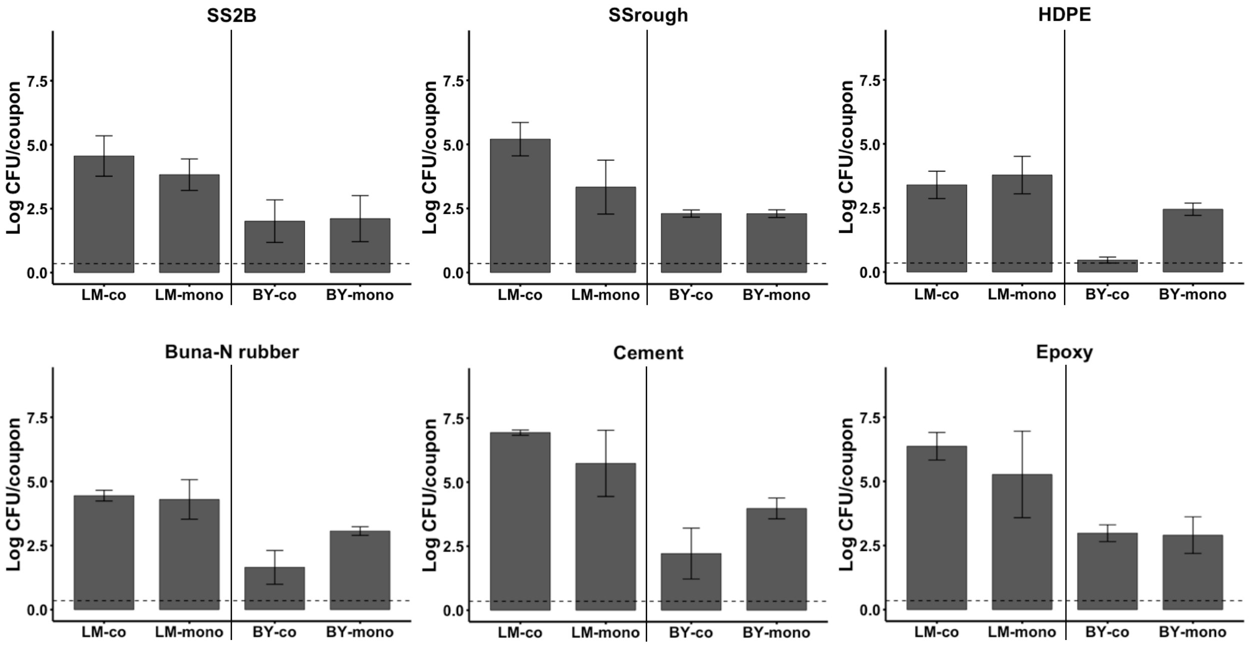

Fig S.1. Comparison of survivors (LM: *Listeria monocytogenes*; BY: Black yeast, also *Exophiala* spp.) from monoculture (mono) and co-culture (co) following the least intense treatment combination: 0.6 mL/L sanitizer and 50 rpm for 30 s at 23.9°C (0.015 Pa shear stress). The limit of detection is at 0.35 Log CFU/coupon (dashed line).

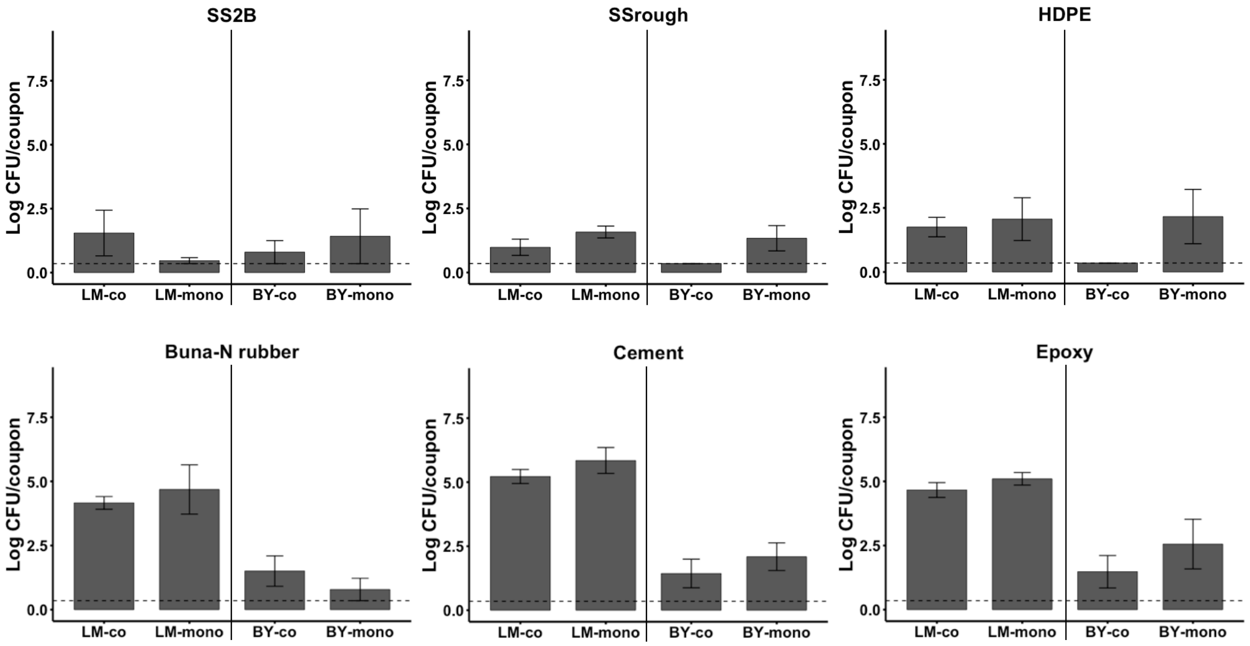

Fig S.2. Comparison of survivors (LM: *Listeria monocytogenes*; BY: Black yeast, also *Exophiala* spp.) from monoculture (mono) and co-culture (co) following the most intense treatment combination: 2.4 mL/L sanitizer at 900 rpm for 5 min at 23.9°C (4.99 Pa shear stress). The limit of detection is at 0.35 Log CFU/coupon (dashed line).

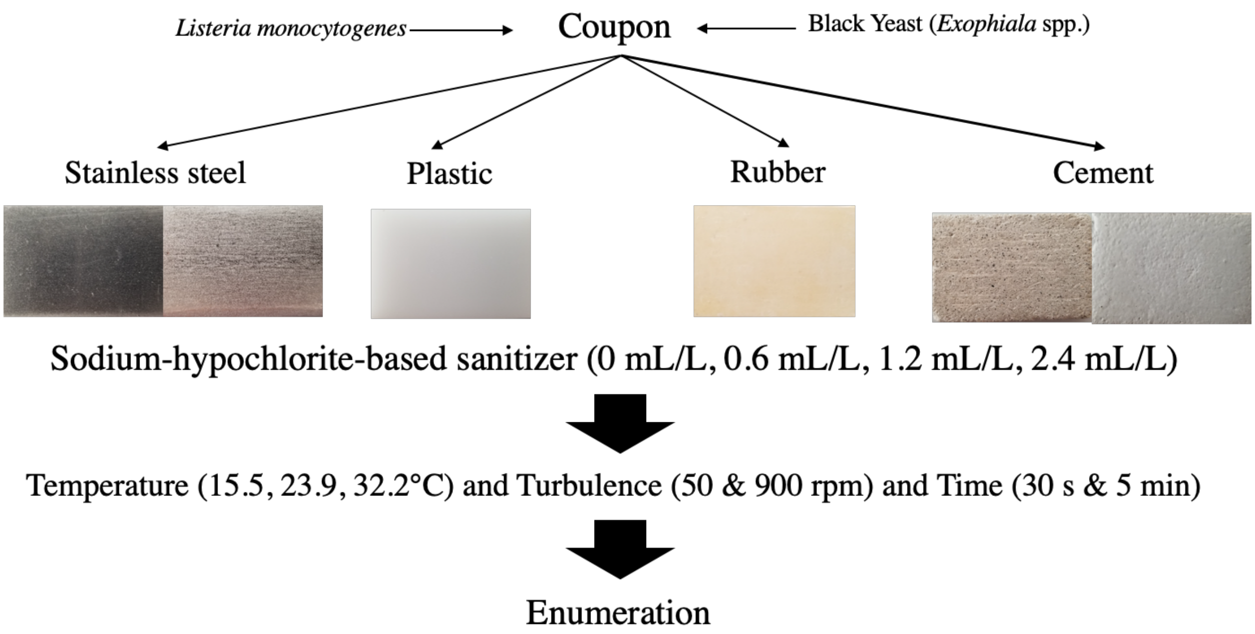

Fig S.3. Flow chart

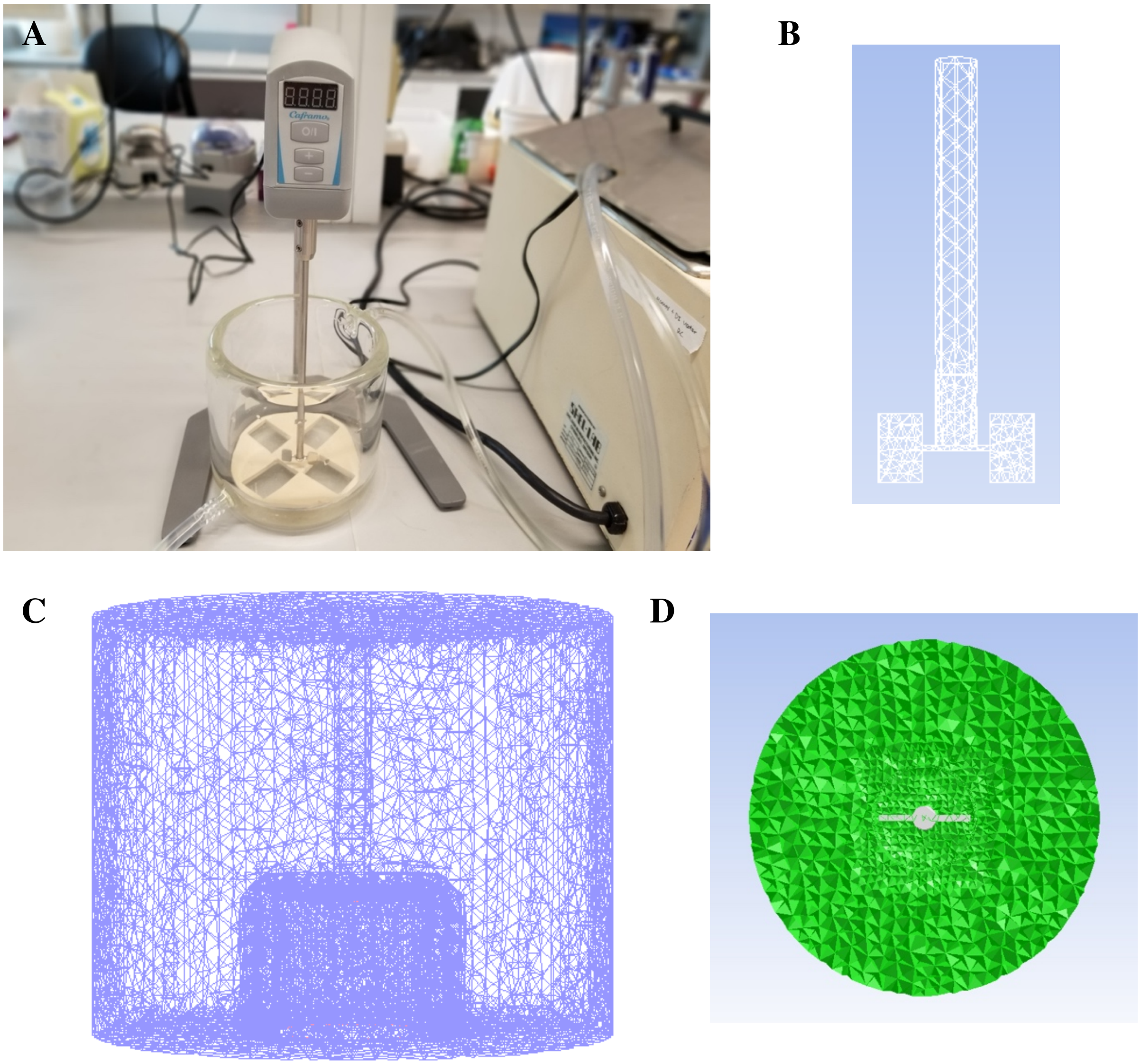

Fig. S.4. Bioreactor (A) and the 3-D Mesh of the stirred vessel bioreactor: (B) side view of the impeller, (C) side view of the stirred vessel, (D) top view of the stirred vessel. Figures S4B-D were taken from Fan, 2018.

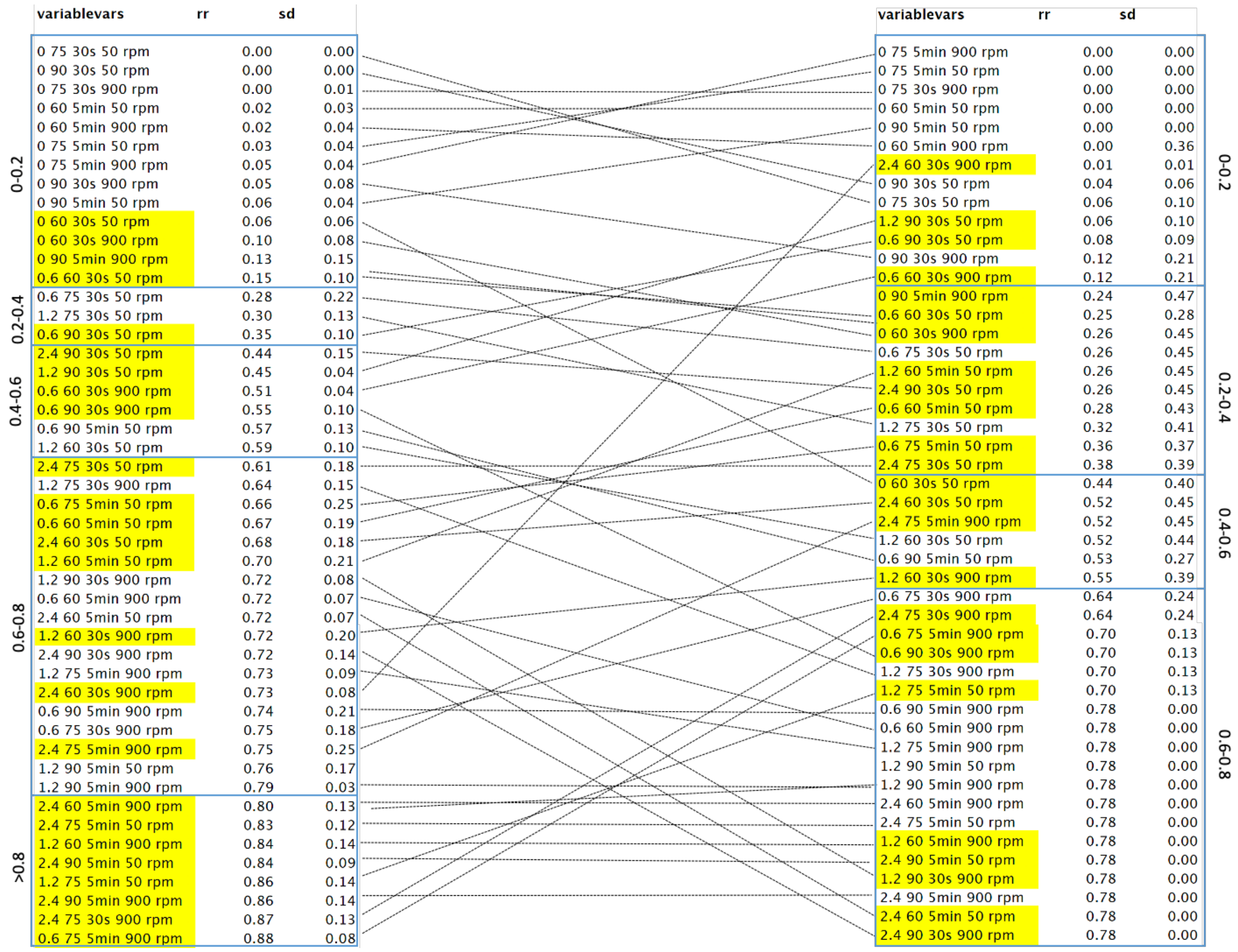

Fig. S.5. Tanglegram comparing treatment efficacy (relative reduction) between *Listeria monocytogenes* and *Exophiala* spp. on SS_{2B} coupons (treatment combination: sanitation concentration (mL/L), water temperature (°F), contact time, rotational fluid velocity). Highlighted treatment combination: LM and BY relative reductions belong to different blocks.
